# Deciding on Interferon-Free Treatment for Chronic Hepatitis C: Updating Liver Stiffness Cut-Off Values to Maximize Benefit

**DOI:** 10.1371/journal.pone.0164452

**Published:** 2016-10-10

**Authors:** Agostino Colli, Mirella Fraquelli, Daniele Prati, Alessia Riva, Alessandra Berzuini, Dario Conte, Alessio Aghemo, Massimo Colombo, Giovanni Casazza

**Affiliations:** 1 Department of Internal Medicine, Ospedale A. Manzoni, Lecco, Italy; 2 Gastroenterology and Endoscopy Unit, Fondazione IRCCS Ca’ Granda Ospedale Maggiore Policlinico, Milan, Italy; 3 Department of Transfusion Medicine and Hematology, Ospedale A Manzoni, Lecco, Italy; 4 Gastroenterology and Hepatology Unit, Fondazione IRCCS Ca’ Granda Ospedale Maggiore Policlinico, Milan, Italy; 5 Department of Biomedical and Clinical Sciences, Università degli Studi di Milano, Milan, Italy; Yonsei University College of Medicine, REPUBLIC OF KOREA

## Abstract

**Introduction:**

In a perspective of economic constraints the prioritizing of patients to IFN-free regimens is mainly based on the determination of liver stiffness by transient elastography (TE). Being a continuous variable the interpretation of TE results requires the identification of cut-off values, to date set to maximize diagnostic accuracy even if such values should be better based on more helpful outcome prediction endpoints.

**Aim:**

To define the TE cut-off values in different clinical scenarios, including new IFN-free regimens, and to balance the clinical benefits versus harms in treated and untreated patients.

**Methods:**

We assessed the accuracy of TE in staging 728 consecutive HCV patients and the distribution of TE values in 1,001 blood donors. Ten experts quantified the expected harm/benefit ratio for 6 scenarios resulting from 2 stages of liver disease (F≥2 or F≥3) and 3 treatment regimens: PEGIFN+ribavirin, PEGIFN+RBV+first-generation protease inhibitor, and IFN-free regimens. The optimal TE cut-off values were identified using the Metz equation.

**Results:**

The estimated mean expected harm/benefit ratio for IFN-free regimens was 1/8.3 in patients with F≥2 and 1/10 in those with F≥3. The resulting optimal cut-off values were respectively 4.5 kPa with sensitivity at 99% and specificity at 12%, and 6.8 kPa with sensitivity at 94% and specificity at 41%. These cut-off values are lower than those maximizing accuracy and allow to reduce the number of false negative results.

**Conclusions:**

The optimal TE cut-off values to prioritize patients for IFN-free regimens, are sensibly lower than those used to maximize diagnostic accuracy.

## Introduction

The limitation of resources for most national health services precludes the antiviral treatment of all HCV patients and leads to the need of prioritizing treatment mainly for those patients at highest risk for disease complications.

Especially for IFN-free regimens, which are highly effective and safe but particularly expensive, the prioritization is mainly decided on the basis of the stage of liver fibrosis, assessed by liver histology, which in the last few years has been almost completely substituted by the assessment of LS via transient elastography.

In order to interpret the results of transient elastography and identify whether the disease is or is not present (i.e., the stage of liver fibrosis), TE being a continuous variable, a cut-off should be chosen, habitually on the basis of diagnostic accuracy. In fact, diagnostic studies usually concentrate on diagnostic accuracy, i.e. the concordance between test results and a reference standard, yet this approach does not necessarily translate into better clinical outcomes, which can only be assessed after a new test has been introduced into clinical practice [1]. If test results are expressed on a continuous scale, identifying the presence or absence of the target disease is based on a cut-off value usually chosen on the basis of accuracy (i.e. the best balance between sensitivity and specificity). However, an alternative and more clinically oriented approach would be to consider the downstream effects of testing in terms of false negative and false positive results: such an approach would identify those patients with the minimum degree of “abnormality” requiring treatment. In this scenario, the disease would be regarded in terms of risk prediction, and diagnosis would play a decision-making role [2].

The measurement of liver stiffness (LS) by means of transient elastography (TE) enables the staging of liver disease severity, particularly for the most frequent chronic infection, i.e. the hepatitis C virus (HCV), and supports the making of decisions on its possible treatment [3] as LS is a validated indicator of liver disease severity, which is a criterion to access antiviral treatments. The currently used TE cut-off values for ‘significant’ and ‘severe’ liver disease are respectively 7.65 and 9.6 kPa, following their maximal concordance with the findings of the liver histology reference standard [4–7]. However, the TE cut-off values for maximizing the possible treatment benefits potentially change depending on the available therapeutic options: therefore it becomes necessary to define new values for supporting the eventual administration of interferon (IFN)-free anti-HCV regimens.

## Materials and Methods

### HCV patients

With the aim of defining the accuracy of TE-based LS measurements, our study considered all of the consecutive HCV-positive patients who underwent a liver biopsy for the staging of chronic hepatitis C at the A.M. & A. Migliavacca Center for Liver Disease in Milan (Italy) between October 2005 and October 2014. The inclusion criteria were: serum alanine aminotransferase (ALT) levels to be persistently or intermittently >1.5 times the upper reference limit in the presence of serum markers of HCV infection; patients with ascites were excluded. The study protocol was approved by the Ethics Committee of Fondazione IRCCS Ca’ Granda Ospedale Maggiore Policlinico, Milan (ref. 78/2005). The patients fulfilling the inclusion criteria were enrolled after obtaining their written informed consent.

The patients underwent TE on the same day but before liver biopsy. The right lobe of the liver was targeted with access through an intercostal space, while the patient was in a dorsal decubitus position with his/her right arm in maximal abduction. A portion at least 6 cm thick and free of large vessels was identified under FibroScan^®^ ultrasound (US) guidance. Only the examinations with at least 10 valid measurements and a success rate of at least 60% (calculated as the ratio between the number of valid measurements and the total number of measurements) were considered reliable. The median of all the successful measurements was expressed in kilo Pascal (kPa) and considered representative of LS in a given patient, but only if the interquartile range (IQR) of all the valid measurements was less than 30% of the median value [5]. The liver biopsies were carried out under US guidance, using a 16 Gauge modified Tru-Cut needle (Biomol, HS, Rome, Italy), to obtain samples longer than 2.5 cm; in the case of shorter samples a second biopsy was performed. Liver fibrosis and necroinflammatory activity were semiquantitatively evaluated by METAVIR scoring [8, 9]. Mean and median values, standard deviations (SD) and ranges were calculated for descriptive purposes.

### Healthy controls

In order to set the upper reference limits for LS, we examined a consecutive series of blood donors attending the Department of Transfusion Medicine and Hematology at the A. Manzoni Hospital in Lecco (Italy), where behavioral, clinical and laboratory examinations of about 15,000 candidate blood donors are carried out every year. The study was proposed to a random sample of donors (those attending the center to donate blood on Mondays, Tuesdays and Wednesdays) with the aim of enrolling 1,000 subjects who had no medical or behavioral contraindication to blood donation and were negative to the serological and nucleic acid testing (NAT) markers of HBV, HCV and HIV-1/2 and to a hemagglutination test for syphilis. The laboratory panel included: a complete blood count, serum liver biochemistries, and glucose, cholesterol and triglyceride concentration. The patients’ weight and height were measured in order to calculate their body mass index (BMI), and a 24.9 kg/m^2^ BMI was considered the upper limit for healthy weight. LS was assessed using a FibroScan^®^ unit (Echosens, Paris, France) and the aforementioned procedure. The results were expressed as median values and 5^th^ 25^th^ 75^th^ and 95^th^ percentiles.

### Harm/benefit ratio estimation

A questionnaire was submitted to 10 liver units in Italy (3 in Milan, and one each in Lecco, Bergamo, Parma, Bologna, Naples, Messina, and Palermo) requesting their clinical (or holistic) assessment of the benefit/harm (B/H) ratios of three possible anti-HCV treatments for two different stages of chronic hepatitis C (genotype 1), i.e. pegylated IFN [PEGIFN] + ribavirin [RBV], PEGIFN + RBV + first-generation protease inhibitor [PI], and IFN-free regimens. The word “harm” was used in order to ensure that the requested judgment would take no financial costs into account. The term” benefit” refers to the subjective evaluation of how to prevent cirrhosis development, its complications and liver-related death.

The questionnaire was preceded by the sharing of background information defining benefit and harm, and including two non-hepatological clinical examples (pulmonary embolism and sore throat). “Benefit” was defined as the net advantage of being treated in the presence of the target disease (i.e. a true positive case), while “harm” was defined as any damage due to treatment in the absence of the target disease (i.e. a false positive). The B/H ratio was expressed as a numerical ratio (without units) representing the relative weight of the advantages vs. disadvantages of treatment. [10]

### Statistical methods

Receiver operating characteristic (ROC) curves of LS in the two target conditions (severe and significant liver fibrosis) were obtained from the available cohort of HCV patients using a bi-normal model that assumed the normal distribution of log-transformed LS values. The overall accuracy of the LS measurements was estimated using the area under the ROC curve (AUROC). Subsequently, and as suggested by Metz [11], the optimal cut-off value (i.e. the value that maximizes the overall expected benefit) for each of the six scenarios was identified as the point on the ROC curve at which the slope of the curve is equal to:

$$\frac{\text{H}}{\text{B}}\cdot \frac{1-p(D)}{p(D)}$$

where H is harm, B benefit and p(D) the prevalence of the target condition. The cut-off values corresponding to the maximum value of Youden’s index (i.e. maximum sensitivity+specificity-1) were also identified [12].

A summary table was also produced showing the effects of different cut-off values in a virtual cohort of 1,000 patients with the same prevalence of severe fibrosis as in our sample.

## Results

### Definition of LS normal values

LS measurement was proposed to 1,014 blood donors: 1,001 accepted and were enrolled; valid measurements were obtained in 977 (97.6%, 95% CI 96.6%‒98.5%). Table 1 shows the distribution of LS values by gender: the 50^th^ and 95^th^ percentiles were respectively 4.4 kPa and 7.6 kPa.

**Table 1:** Distribution of liver stiffness measurements in 1,001 blood donors.

Gender	No. of subjects		Liver stiffness values (kPa)				
	*Total*	With valid measurements*	*Percentiles*				
			*5*^*th*^	*25*^*th*^	*50*^*th*^	*75*^*th*^	*95*^*th*^
**Males**	581	575	3.2	4.0	4.6	5.6	7.8
**Females**	420	402	2.8	3.4	4.1	4.8	7.4
**Overall**	1,001	977	3.0	3.8	4.4	5.3	7.6

*Invalid measurements were obtained in 24 subjects (2.4%, 95% CI 1.4%‒3.4%): 6 males (1.03%, 95% CI 0.4%‒2.2%) and 18 females (4.1%, 95%CI 2.4%‒6.4%).

Forty-eight donors showed values above the upper 95^th^ percentile: the presence of liver disease was excluded in 31 (68%) on the basis of a clinical work-up consisting of a physical examination, liver US, liver enzyme monitoring, the measurement of anthropometric indices, and re-evaluation of their medical history. Seventeen individuals (32%) had signs of chronic liver disease, including steatosis, increased liver enzyme levels, obesity, and glucose intolerance. All but two people had BMI >25 kg/m^2^.

### Analysis of the LS values in patients with chronic HCV-related liver disease

In the cohort of 787 eligible patients with chronic HCV-related liver disease, six people refused to undergo liver biopsy or had some contraindication, while 781 patients consecutively underwent TE scanning followed by ultrasound-guided liver biopsy during the same session. The 728 patients who satisfied the study criteria, were 419 males and 309 females, with a mean age of 51 years, a mean BMI of 24.3 kg/m^2^, and a mean LS value of 10.8±8.9 kPa (range 2.5–75 kPa). Table 2 summarizes the patients’ main clinical and laboratory data. Examination by TE was unsuccessful in 53 patients (7%) because of stiffness measurement failure (n = 8) or unreliability (i.e. a success rate <60% and/or IQR >30% of validated measurements, n = 45).

**Table 2:** Demographic and clinical characteristics of HCV patients concomitantly undergoing TE and liver biopsy assessments of liver stiffness.

Variables	Patients (n = 728)
Males/females	419/309
Mean age ± SD (range), years	51±12 (23–71)
Mean BMI ± SD, kg/m^2^	24.3±3.6
Mean ALT ± SD (range), IU/L (n.v. <38)	98±95 (14–567)
Mean AST± SD (range), IU/L (n.v. <38)	92±89 (10–678)
Mean GGT± SD (range), IU/L (n.v. <50)	68±76 (7–546)
Platelet count± SD (range), 10^9^/L	210±73 (51–638)
Liver stiffness± SD (range), kPa	10.8±8.9 (2.5–75)
F ≥2 (prevalence)	387 (53.2%)
F ≥3 (prevalence)	222 (30.5%)

All the liver specimens obtained by liver biopsy satisfied the predefined reference standard adequacy criterion, i.e. 12 complete portal tracts.

**Fig 1:**
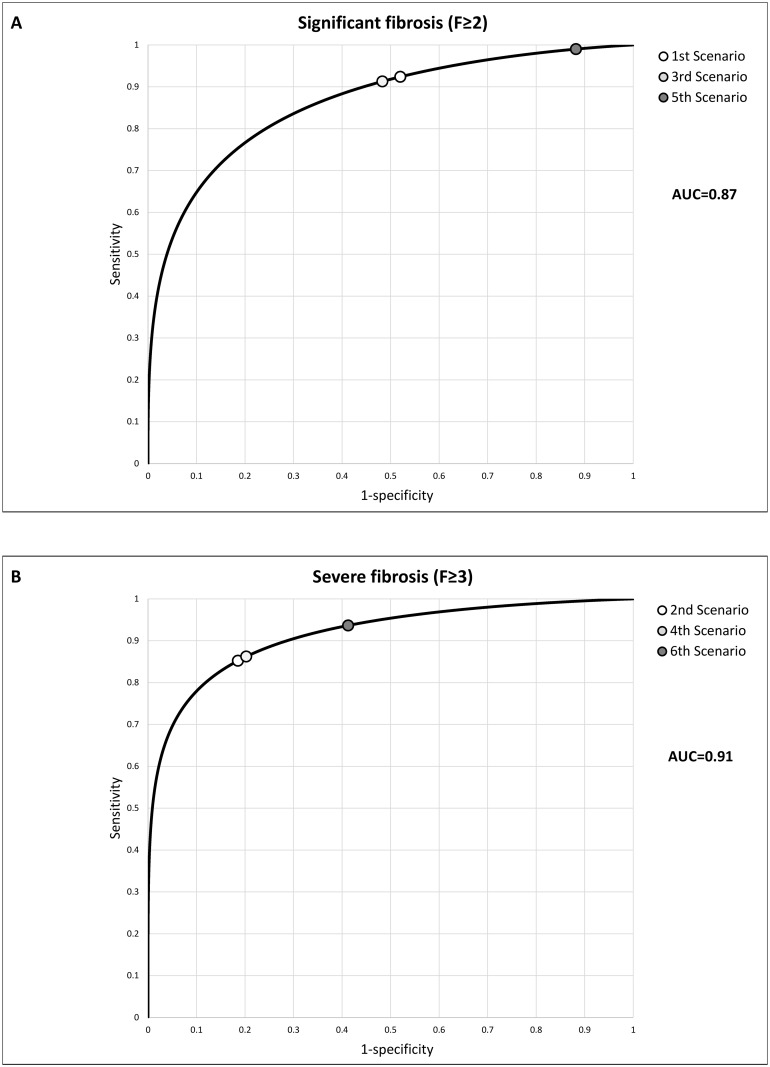
Receiver operating characteristic (ROC) curves, areas under the curve (AUROC), and cut-off values in the different scenarios for significant fibrosis (Panel A) and severe fibrosis (Panel B).

Fig 1 shows the ROC curves of the overall diagnostic accuracy of the LS measurements for the diagnosis of significant (F≥2) and severe fibrosis (F≥3): the AUROCs were respectively 0.87 and 0.91.

### Estimation of H/B ratios

All the 10 hepatology centers receiving the questionnaire, completed and returned it. Table 3 shows the H/B ratios that each center assessed for each scenario and their mean values.

**Table 3:** Estimates benefit/harm ratios for the six scenarios estimated by 10 Italian hepatology centers.

	1^st^ F≥2 PEG-IFN + RBV	2^nd^ F≥3 PEG-IFN + RBV	3^rd^ F≥2 PEG-IFN + ribavirin + first-generation protease inhibitors	4^th^ F ≥3 PEG-IFN + RBV + first-generation protease inhibitors	5^th^ F ≥2 IFN-free regimens	6^th^ F≥3 IFN-free regimens
**Palermo**	2	3	3	4	8	10
**Bergamo**	6	7	4	7	10	10
**Milan 1**	1.33	3	1	2.25	4	9
**Naples**	2	3	3	4	5	10
**Parma**	5	3	2	5	9	10
**Milan 2**	6	6	7	8	10	10
**Messina**	2	3	3	4	9	8
**Lecco**	2.5	2.75	2	1.44	17	19
**Milan 3**	1.75	5	1.4	3.3	7	10
**Bologna**	3	1.5	1.5	2	4	4
**Mean value**	**3.1**	**3.7**	**2.8**	**4.1**	**8.3**	**10**

### Definition of the LS cut-off value maximizing the expected benefit

Taking into account the prevalence of the target disease (significant or severe fibrosis) and the mean H/B ratios, the corresponding optimal cut-off –value was calculated for each scenario as described in Methods. Table 4 shows the mean H/B ratios and the six different optimal cut-off values. The optimal cut-off points in the ROC curves for significant and severe fibrosis are shown in Fig 1. In Table 5 with regard to the 5^th^ and 6^th^ scenario we summarized the accuracy of three different LS cut-off values: i.e. the value calculated to maximize benefit [11], the value in use (derived from a meta-analysis of accuracy) [4], and the value derived *post hoc* on the basis of our results using the maximum of Youden’s index.

**Table 4:** Harm/benefit ratios and corresponding liver stiffness cut-off values in all the scenarios.

	1^st^ F≥2 PEG-IFN+RBV	2^nd^ F≥3 PEG-IFN+RBV	3^rd^ F≥2 PEG-IFN+RBV+PI	4^th^ F≥3 PEG-IFN+RBV+PI	5^th^ F≥2 IFN-free	6^th^ F≥3 IFN-free
H/B ratio*	1/3.1	1/3.7	1/2.8	1/4.1	1/8.3	1/10
**LS values (kPa)**	6.1	9.4	6.4	9.2	4.5	6.8

*mean value of the 10 liver units.

**Table 5:** Liver stiffness measurement. Effects in a virtual cohort of 1,000 patients with chronic hepatitis C in the 5^th^ scenario (significant fibrosis and interferon-free regimens) and 6^th^ scenario (severe fibrosis and interferon free regimens).

**5**^**th**^ **SCENARIO F≥2 (significant fibrosis)**								
	Cohort n =	Prevalence F≥2	Sensitivity	Specificity	TP	FP	FN	TN
Benefit cut-off (4.5 kPa)	1000	53.2%	0.990	0.118	526	413	6	55
Cut-off in use (7.65 kPa)	1000	53.2%	0.864	0.645	459	167	73	301
Youden (9.3 kPa)	1000	53.2%	0.741	0.827	394	81	138	387
**6**^**th**^ **SCENARIOF≥3 (severe fibrosis)**								
	Cohort n =	Prevalence F≥3	Sensitivity	Specificity	TP	FP	FN	TN
Benefit cut-off (6.8 kPa)	1000	30.5%	0.937	0.413	285	408	20	287
Cut-off in use (9.6 kPa)	1000	30.5%	0.82	0.859	250	98	55	597
Youden (10.0 kPa)	1000	30.5%	0.801	0.881	244	83	61	612

Benefit cut-off: cut-off defined according to the Metz method.

Cut-off in use: cut-off derived from previous studies.

Youden; cut-off derived according to Youden’s Index.

Sensitivity and specificity assessed using liver biopsy as the reference standard.

TP: true positives, FP: false positives, FN: false negatives, TN: true negatives.

#### Significant fibrosis (F≥2)—5^th^ scenario

Considering a virtual cohort of 1,000 patients, and assuming the same prevalence of fibrosis F≥2 (53.2%) as that observed in our cohort of patients with chronic hepatitis C, and using the cut-off value of 4.5 kPa maximizing the benefit of IFN-free regimen, we would obtain 6 false negative and 413 false positive results. With the usual cut-off value of 7.65 kPa we would have obtained 73 false negative and 167 false positive results. Finally, with the cut-off value at 9.3 kPa obtained maximizing Youden’s index we would obtain 138 false negative and 81 false positive results (Table 5).

#### Severe fibrosis (F≥3)—6^th^ scenario

Considering a virtual cohort of 1,000 patients and assuming the same prevalence of fibrosis F≥3 (30.5%) as that observed in our cohort of patients with chronic hepatitis C, and using the cut-off value of 6.8 kPa maximizing the benefit of IFN-free regimen, we would obtain 20 false negative and 408 false positive results. Whereas with the usual cut-off of 9.6 kPa we would have obtained 55 false negative and 98 false positive results. Finally, with the cut-off at 10.0 kPA obtained maximizing Youden’s index we would obtain 61 false negative and 83 false positive results (Table 5).

## Discussion

The access of patients to antiviral treatment, especially after the introduction of the highly effective but extremely expensive IFN-free regimens, is mainly based on the assessment of liver fibrosis stage.

The consideration that a good level of cost effectiveness is obtained treating patients at an early stage of disease [13] and that chronic HCV infection also causes significant extra-hepatic morbidly [14], leads to the requirement of redefining the TE cut-off, switching from merely diagnostic criteria to disease-outcome-predicting endpoints, this implying an extensive hepatologic clinical judgment.

TE cut-off values actually serve the purpose of maximizing diagnostic accuracy according to Youden’s index, which is based on the sum of true positive and true negative results [12]. In this scenario, the downstream clinical consequences of testing are not explicitly evaluated, and the effect of the false positive and false negative results is implicitly considered equivalent. However, when there is the aim of setting a cut-off value in order to support clinical decision-making, the consequences of false results (i.e. from treating subjects who do not have the target condition and or from not treating subjects who have it) should be weighed, and the cut-off value should vary depending on the treatment options to avail.

Using chronic hepatitis C as an example, we have showed that the diagnostic test cut-off values can be defined with the aim of maximizing the benefit of treatment rather than overall diagnostic accuracy. The decisions concerning the treatment of patients with chronic hepatitis C are driven by the stage of fibrosis and liver disease severity [3, 13, 15–19] which can be accurately and safely assessed by measuring LS as an alternative to percutaneous liver biopsy. Furthermore, the results can be expressed as continuous variables, thus enabling the categorization of multiple cut-off values, which can be optimized for different clinical scenarios [8]. We have obtained two LS ROC curves for diagnosing significant and severe fibrosis/liver disease in patients with chronic hepatitis C, using liver histology as the reference standard. We have then identified the cut-off values maximizing treatment benefit in 6 therapeutic scenarios (Fig 1) by taking into account the harm/benefit ratio of the treatment defined by a group of expert clinicians and the prevalence of the target condition [6].

In the scenarios of IFN-based treatment, which is less effective and more harmful than IFN-free regimens, we have identified cut-off values that are close to those currently in use as calculated by maximizing diagnostic accuracy (7.65 kPa for significant fibrosis and 9.6 kPa for severe fibrosis) [4]. However, when the scenarios changed to those of IFN-free regimens, the optimal cut-off value decreased to 6.8 kPa when the aim was to treat patients with severe fibrosis, this figure being close to the upper reference limit obtained on the basis of the distribution of LS values in healthy blood donors (7.6 kPa) (Table 2). This indicates that, when highly effective and safe treatment options are available, clinicians favor sensitivity over specificity and end up electing for treatment patients with any degree of fibrosis. Furthermore, when the threshold was lowered in order to include patients with significant fibrosis, the optimal cut-off value (4.5 kPa) was close to the 50^th^ percentile of the healthy donors, thus indicating that all people are potentially treated regardless of their LS values because the possibility of treating carriers without any degree of fibrosis is clinically acceptable.

As expected, our data shows that, in the case of a very low H/B ratio such as that of IFN-free regimens, the penalty from being false positive is more acceptable than that from being false negative. Furthermore, if the H/B ratio is close to one (as in the case of the two obsolete treatments), the same weight is attributed to false positive or negative test results, and the same cut-off value can maximize both accuracy and treatment benefit.

The application of the new cut-off values in a virtual cohort of 1,000 patients with chronic hepatitis C and a 30% prevalence of severe fibrosis increases the number of true positive results to 35 (with the consequent benefit of IFN-free treatment) at the expense of 310 more false positive results: i.e. the penalty from being false negative clearly outweighs the penalty from being false positive (Table 4). Similarly, in the case of significant fibrosis, the new cut-off value would lead to IFN-free treatment for further 313 patients (67 true and 246 false positive).

The estimated cut-off values obtained using Metz’s equation [11] has not only depended on the H/B ratio, but also on the prevalence of the target condition (severe or significant fibrosis) as a different prevalence leads to a different number of false results: in the case of a higher prevalence, there are usually more false negative results, and in the case of a lower prevalence, there are likely to be more false positive results. However, the prevalence of severe and significant fibrosis among the patients with chronic hepatitis C of this study appears to be quite similar to the rates observed also by other centers [9–12], and therefore the LS cut-off estimates are mainly due to the H/B ratios of the treatment options.

One major advantage of the clinically oriented approach described in this study is that any center can set its own cut-off value to reflect the prevalence of the target condition and balance the expected rates of false positive and negative results against treatment efficacy; the values could be even adjusted to an individual patient’s characteristics or preferences [20]. For example, a patient’s age which has not been taken into account in the 6 scenarios presented here for the sake of simplicity, is an important factor related to life expectancy and benefit estimation, and can be included in the model. Furthermore, the average of the H/B ratios estimated by different centers can help define homogenous rules against resource constraints, as in the case of very expensive IFN-free regimens. This approach has been suggested over 15 years ago for tuberculosis testing, mammography and palpation for breast cancer, Shotz tonometry for glaucoma, etc., but it has been very rarely applied in practice [10, 11]. There is a widespread perception that there needs to be a link between diagnosis and treatment, and it is generally accepted that tests should not be used unless their results change patient management [21]. The measurement of LS in patients with chronic hepatitis C seems to satisfy these requirements, and the same method of defining cut-off values can also be applied to other validated non-invasive tests for staging hepatitis C as FibroTest^®^, platelet ratio index (APRI) and FIB-4 or other liver diseases such as chronic hepatitis B.

A possible limitation of our study is that as healthy controls we have used a population of blood donors in whom the presence of latent liver disease cannot be absolutely excluded even against their repeated normal blood tests.

Another limitation is that we have compared the TE LS values with the histologic findings of liver biopsies, which are currently considered an imperfect reference standard as they lead to a false negative rate of 20% in the case of a diagnosis of severe fibrosis [23–27]. It is possible that some of the LS results were classified as false positive whereas they may be in fact true positive and false negative at biopsy histology, meaning that the specificity of LS measurements has been possibly underestimated despite our efforts to reduce biopsy sampling errors by accepting adequately sized specimens only. However, in order to counteract the possible underestimation of this specificity, the recommended cut-off values would have been even lower. Of note, we have used inclusion criteria requiring ALT elevation >1.5 ULN, which is no more required.

A further possible shortcoming of this study is that the estimated H/B ratios (Table 3) were obtained on the basis of the holistic clinical judgement of the specialists working in the participating centers, and are therefore a potential source of heterogeneity. However, it is unlikely that the alternative approach of building a more formal cost/benefit analysis would have addressed such an issue because there is little data concerning the natural history of hepatitis C or the extent of benefit or harm arising from treatment [22]. Interestingly, in the literature there are several examples supporting clinical judgment as a valid approach to complex clinical decisions [28, 29].

In conclusion, we suggest that a “*one cut-off value fits all”* approach is not appropriate, especially when the results of a single test are the major driver of a clinical decision, and that cut-off values should vary depending on the H/B profile of the proposed/available treatments. Given that for the IFN-free regimens of chronic hepatitis C treatment the benefits largely exceed the harms, the LS values justifying treatment initiation are lower than those computed on the basis of diagnostic accuracy, independently of the liver fibrosis score.

Specifically, our study emphasizes that in the setting of IFN-free regimens the values of LS indicating the need to start antiviral treatment are sensibly lower than those used only to maximize diagnostic accuracy as usually incorporated in the clinical guidelines. Thus, in the context of economic constraints the decision to treat or not to treat should be based on clinical considerations and the assessment of LS should be re-interpreted. From a practical viewpoint, when limited resources preclude the treatment of all patients, independently from the severity of the disease, an appropriate prioritization strategy is needed. Compared to the current cut-off set at 10 kPa chosen in most European countries, a new cut-off value defined on the basis of the expected harm/benefit ratio would reduce the number of inappropriately untreated patients, i.e. the false negative results.

On the other hand, in those national health system enjoying a more favorable level of resources and where the access to IFN-free regimens is broader, including also patients with mild to moderate, or absent fibrosis, LS determination by TE is not helpful or justified.

## Abbreviations

B/H ratio: benefit/harm ratio
IFN-free regimens: interferon-free regimens
PEGIFN+RBV+first-generation: peginterferon + ribavirine first-generation protease inhibitor
ROC: receiver operating characteristic curve
TE: transient elastography
